# Association between home smoking and e-cigarette use restrictions and concurrent use behaviors among dual users of cigarettes and e-cigarettes

**DOI:** 10.18332/tid/209376

**Published:** 2025-09-25

**Authors:** Vuong V. Do, Jidong Huang, Terry F. Pechacek, Claire A. Spears, David L. Ashley, Carla J. Berg, Scott R. Weaver

**Affiliations:** 1 Center for Tobacco Control Research and Education, University of California San Francisco, San Francisco, United States; 2 Department of Health Policy and Behavioral Sciences, School of Public Health, Georgia State University, Atlanta, United States; 3 Department of Prevention and Community Health, Milken Institute School of Public Health, George Washington University, Washington, United States; 4 Department of Population Health Sciences, School of Public Health, Georgia State University, Atlanta, United States

**Keywords:** cigarettes, e-cigarettes, smoke-free home policy, home smoking restrictions, home e-cigarette restrictions

## Abstract

**INTRODUCTION:**

Limited data exist on how home smoking and e-cigarette use restrictions influence patterns of cigarette and e-cigarette use among individuals who use both products. This study examined the association between home restrictions and the use of cigarettes and e-cigarettes among dual users of these two products.

**METHODS:**

We conducted a secondary analysis of data from the Adult Consumers of Tobacco Study (ACTS), an online, nationwide survey administered during 2020–2021. A sub-sample of 250 dual users of cigarettes and e-cigarettes was included in this analysis. Outcome variables were concurrent use behaviors, categorized as predominant smokers (daily smoking, e-cigarette use some days), equivalent users (either daily or some days use of both products), and predominant e-cigarette users (daily e-cigarette use, smoking some days), as well as e-cigarette use frequency (daily vs some days). Primary explanatory variables were home smoking and e-cigarette use restrictions (both categorized as complete, partial, or no restrictions). Multivariable logistic regression was used to examine associations between restrictions and concurrent use behaviors.

**RESULTS:**

Nearly half (46.8%) of dual users reported having complete smoking restrictions in their homes, complete e-cigarette use restrictions (12.8%), and complete restrictions on both (11.2%). Dual users reported having complete home smoking restrictions (vs no restrictions) were more than two times more likely to be predominant e-cigarette users versus equivalent users or predominant smokers (AOR=2.60; 95% CI: 1.30–5.30), after controlling for home e-cigarette use restrictions and other covariates. Neither partial smoking restrictions nor partial e-cigarette use restrictions were associated with concurrent use behaviors.

**CONCLUSIONS:**

Small proportions of dual users reported having complete smoking and e-cigarette use restrictions adopted in their homes, suggesting a need for promoting the adoption of such restrictions. Moreover, how and why home smoking and e-cigarette use restrictions differentially impact use behaviors warrant additional investigation.

## INTRODUCTION

The use of e-cigarettes is popular among US adults who smoke cigarettes, with almost 14% of them using e-cigarettes concurrently^1^. One of the important reasons for e-cigarette use among adults who smoke is their purported role in helping them quit cigarette smoking^2^. A recent Cochrane systematic review and meta-analysis indicated moderate-certainty evidence that e-cigarettes with nicotine increase quit rates compared with e-cigarettes without nicotine and nicotine replacement therapy^3^. However, the role of e-cigarettes in smoking cessation under real-world use conditions is still inconclusive^4^. In addition, a few studies found that other commonly reported reasons to use e-cigarettes among people who smoke include that e-cigarettes were perceived as less harmful than cigarettes to users and bystanders and that e-cigarettes can be used when/where smoking is not allowed^5^. Regardless of the reasons to use, dual use of cigarettes and e-cigarettes can only contribute positively to population health when such behavior is transitory and followed by a complete switch to e-cigarettes or a complete quit of tobacco and nicotine products^6^.

Prolonged dual use of cigarettes and e-cigarettes is not associated with improved health outcomes. Indeed, some studies have found that dual use of cigarettes and e-cigarettes is associated with a higher risk of cardiovascular disease than only smoking cigarettes^7,8^. Dual users of cigarettes and e-cigarettes exhibit heterogeneous behaviors of use^9^ that may be distinct for the two products and may contribute to different cessation outcomes and/or health risks. Thus, it is important to understand subgroups of dual users’ smoking and e-cigarette use behaviors rather than considering all dual users as a homogenous group.

Smoke-free laws are an effective policy tool to protect non-smokers from secondhand smoke, increase cessation among people who smoke, and reduce smoking prevalence in the general population^10-12^. In addition, the enactment of smoke-free laws in public places increases the adoption of voluntary home smoking restrictions^13^. Home restrictions on smoking combustible cigarettes (hereafter referred to as smoking) offer important environmental and social controls of smoking behaviors and are associated with longer time-to-first-cigarette upon waking^14^, fewer number of cigarettes per day^15,16^, higher intention to quit smoking^17^, more successful cessation attempts^15^, lower likelihood of relapse after cessation^18^, and reduced smoking initiation^19^. However, little is known about how home smoking restrictions affect e-cigarette use and concurrent use behaviors among dual users of cigarettes and e-cigarettes. Even less is known about home e-cigarette use restrictions, including the extent of their presence and their association with behaviors of concurrent use and cessation behaviors and related outcomes.

Research has shown that cigarettes and e-cigarettes may be substitutes for each other^20,21^. For instance, experiments have shown that when the cost per cigarette puff increased and the price per e-cigarette puff was held constant, individuals purchased fewer cigarette puffs but more e-cigarette puffs^21^. These findings suggest that the adoption of indoor cigarette smoking bans could potentially lead to an increase in e-cigarette use if such bans do not include e-cigarettes. In addition, a few studies showed that including e-cigarettes in indoor smoke-free air policies was associated with reduced e-cigarette use among adults^22,23^.

Given the growing concerns about dual cigarette and e-cigarette use, the potentially significant role of home smoking restrictions and such restrictions for e-cigarette use on the concurrent use behaviors, and the gaps in the literature on these topics, this study aimed to examine the presence of home smoking and e-cigarette use restrictions among dual users of cigarettes and e-cigarettes. It also aimed to analyze the association between home smoking and e-cigarette use restrictions and concurrent use behaviors.

We hypothesized that dual users are more likely to be predominant e-cigarette users (vs predominant smokers) or daily e-cigarette users (vs non-daily e-cigarette users) when they have self-imposed home smoking restrictions, whereas home e-cigarette use restrictions might yield the opposite outcome.

## METHODS

### Study design and sample

Data for this study were drawn from the baseline survey of the Adult Consumers of Tobacco study (ACTS), with 318 adults recruited using targeted social and online media ads. Details about the study design and participant recruitment have been reported elsewhere^24^. Briefly, inclusion criteria were: age ≥21 years, past 60-day cigarette use, and past 30-day new e-cigarette use or re-initiation of e-cigarette use after one or more years of non-use. The survey was administered between December 2020 and October 2021. We conducted a secondary data analysis among a sub-sample of 250 current dual users of cigarettes and e-cigarettes at the baseline survey of the ACTS Study. This study was approved by the Institutional Review Board at Georgia State University (approval number: 365089).

### Outcome variables

Outcome variables were concurrent use behaviors of cigarettes and e-cigarettes and e-cigarette use frequency (every day vs some days). The concurrent use behavior variable was derived from the two questions: ‘Do you now smoke cigarettes?’ and ‘Do you now use electronic nicotine products with nicotine?’ with answer options for both questions being ‘every day’, ‘some days’, or ‘not at all’. Dual use status was trichotomized as predominant smoking (if reported smoking every day and using e-cigarettes some days), equivalent use (smoking and using e-cigarettes every day, or smoking and using e-cigarettes some days), and predominant e-cigarette use (smoking some days and using e-cigarettes every day).

### Explanatory variables

The home smoking restriction variable was derived from the question, ‘Which statement best describes the rules about smoking a combustible cigarette inside your home?’ with response options: 1) ‘It is not allowed anywhere or at any time inside my home’ (complete restrictions); 2) ‘It is allowed in some places or at sometimes inside my home’ (partial restrictions); and 3) ‘It is allowed anywhere and at any time inside my home’ (no restrictions). A similar question regarding restrictions on using e-cigarettes in the home was used as a measure for home e-cigarette use restrictions.

Other independent variables include age, gender, total family annual income, education level, race/ethnicity, smoking intensity (i.e. number of smoking days in the past 7 days, number of cigarettes per smoking days, and time to the first cigarette measured in minutes), e-cigarette use intensity (i.e. number of days using e-cigarettes in the past 7 days), whether other household members own e-cigarettes, and stage of change for quitting smoking (precontemplation, contemplation, preparation). The stage of change for quitting smoking was derived from the quitting smoking intention score on the scale from 0 (no thought of quitting) to 10 (now taking action to quit), with precontemplation stage including participants with scores from 0–2, contemplation stage including scores from 3–7, and preparation stage including scores from 8–10^25^.

### Data analysis

Percentages for those with complete, partial, or no smoking and e-cigarette use restrictions, respectively, were estimated overall and by sociodemographic characteristics, smoking/e-cigarette use intensity, concurrent use behavior, and stage of change for quitting smoking. Bivariate associations between home smoking and e-cigarette use restrictions and participant characteristics were examined using chi-squared tests and Fisher’s exact tests for categorical variables, and Kruskal-Wallis tests for continuous variables. Ordinal and logistic regressions were used to estimate adjusted odds ratios (AOR) of home smoking restrictions and home e-cigarette use restrictions in relation to the outcomes of concurrent use behavior and daily (vs non-daily) e-cigarette use, respectively, adjusting for sociodemographic and other covariates (i.e. time to the first cigarette and other members own e-cigarettes at home). For the ordinal regression models, we confirmed the tenability of the proportional odds assumption. Additional models examined the interaction effects of home smoking restrictions with e-cigarette use restrictions (each dichotomized as any restriction vs no restriction), and likelihood ratio tests were used to assess the overall contribution of the interaction effects to model fit. Four participants who reported non-cis gender and five with missing data (on the time to the first cigarette variable) were excluded from the regression analyses to avoid unstable estimates and convergence issues. We also assessed multicollinearity between variables in the regression models using the variance inflation factor (VIF). All statistical tests were two-sided, and we considered p<0.05 to be statistically significant. Analyses were performed using STATA 19 (StataCorp, 2025)^26^.

## RESULTS

Table 1 presents the adoption of home smoking and e-cigarette use restrictions among dual users of combustible cigarettes and e-cigarettes. Just 11.2% (28/250) of dual users reported having complete restrictions on both smoking and e-cigarette use inside their homes. Home smoking restrictions were more common than e-cigarette use restrictions, with 70% having at least some restrictions on smoking versus about 33% having at least some restrictions on e-cigarette use inside their homes. Among those with complete restrictions on smoking (46.8%), 52.2% reported that they could use e-cigarettes anywhere and anytime inside their homes. A significant association was observed between home smoking restrictions and e-cigarette use restrictions (Pearson χ^2^=44.8, p<0.001). Participants who reported having some e-cigarette use restrictions also tended to have stricter smoking restrictions (polychoric correlation, rho=0.58).

**Table 1:** Home smoking and e-cigarette use restriction status and their bivariate associations, a cross-sectional analysis of data from the Adult Consumers of Tobacco Study 2020–2021 (N=250)

*Home smoking* *restrictions*	*Home e-cigarette use restrictions*				*Chi-squared test of* *association*	*Polychoric* *correlation*
	*Complete* *restrictions*	*Partial* *restrictions*	*No restrictions*	*Total*		
Complete restrictions	28 (23.9)	28 (23.9)	61 (52.2)	117 (46.8)	Pearson χ^2^=44.8 p<0.001	Rho=0.58
Partial restrictions	3 (5.2)	17 (29.3)	38 (65.5)	58 (23.2)		
No restrictions	1 (1.3)	4 (5.3)	70 (93.3)	75 (30.0)		
Total	32 (12.8)	49 (19.6)	169 (67.6)	250 (100)		

Frequency and row % are reported, except the Total column (column %). Polychoric correlation, a measure of correlation between two latent continuous variables, ranges from 0 to 1, where 0 indicates no relationship and 1 indicates a perfect relationship.

Table 2 describes frequencies and percentages for complete, partial, and no smoking and e-cigarette use restrictions by participant sociodemographic characteristics. More than one-third (35.9%) of dual users with a total family annual income <$25000 reported having no smoking restrictions. Among non-Hispanic Black dual users, 43.3% and 86.6% reported having no smoking or e-cigarette use restrictions, respectively. However, no significant associations between home smoking or e-cigarette use restrictions and sociodemographic variables were found.

**Table 2:** Home smoking and e-cigarette use restrictions by participant socioeconomic characteristics, a crosssectional analysis of data from the Adult Consumers of Tobacco Study 2020–2021 (N=250)

*Characteristics*	*Home smoking restrictions*				*Home e-cigarette use restrictions*			
	*Complete* *restrictions* *(N=117)*	*Partial* *restrictions* *(N=58)*	*No* *restrictions* *(N=75)*	*p*	*Complete* *restrictions* *(N=32)*	*Partial* *restrictions* *(N=49)*	*No* *restrictions* *(N=169)*	*p*
**Age** (years)				0.87**^a^**				0.31**^a^**
21–34	45 (51.1)	19 (21.6)	24 (27.3)		7 (7.9)	15 (17.1)	66 (75.0)	
35–44	42 (45.1)	22 (23.7)	29 (31.2)		16 (17.2)	20 (21.5)	57 (61.3)	
≥45	29 (42.7)	17 (25.0)	22 (32.3)		9 (13.2)	14 (20.6)	45 (66.2)	
**Gender**				0.73**^b^**				0.16**^b^**
Male	37 (48.7)	18 (23.7)	21 (27.6)		9 (11.8)	16 (21.1)	51 (67.1)	
Female	77 (45.3)	39 (22.9)	54 (31.8)		23 (13.5)	30 (17.7)	117 (68.8)	
Non-cis	3 (75.0)	1 (25.0)	0 (0)		0 (0)	3 (75.0)	1 (25.0)	
**Total family income** ($)				0.33**^b^**				0.07**^b^**
<25000	40 (43.5)	19 (20.6)	33 (35.9)		11 (12.0)	20 (21.7)	61 (66.3)	
25000–49999	40 (46.0)	21 (24.1)	26 (29.9)		9 (10.3)	14 (16.1)	64 (77.6)	
50000–99999	31 (48.4)	18 (28.1)	15 (23.4)		9 (14.1)	12 (18.7)	43 (67.2)	
≥100000	6 (85.7)	0	1 (14.3)		3 (42.9)	3 (42.9)	1 (14.3)	
**Education level**				0.34**^b^**				0.70**^b^**
Lower than high school diploma	6 (42.9)	5 (35.7)	3 (21.4)		3 (21.4)	2 (14.3)	9 (64.3)	
High school diploma or equivalent	34 (43.0)	15 (19.0)	30 (38.0)		11 (13.9)	12 (15.2)	56 (70.9)	
Some college, no degree	46 (49.5)	19 (20.4)	28 (30.1)		12 (12.9)	22 (23.7)	59 (63.4)	
Bachelor’s or higher	31 (48.4)	19 (29.7)	14 (21.9)		6 (9.4)	13 (20.3)	45 (70.3)	
**Race/ethnicity**				0.62**^b^**				0.30**^b^**
White, non-Hispanic	88 (48.6)	40 (22.1)	53 (29.3)		25 (13.8)	38 (21.0)	118 (65.2)	
Black, non-Hispanic	11 (36.7)	6 (20.0	13 (43.3)		2 (6.7)	2 (6.7)	26 (86.6)	
Hispanic, any race	11 (47.8)	7 (30.4)	5 (21.8)		3 (13.0)	4 (17.4)	16 (69.6)	
Other race, non-Hispanic	7 (43.8)	5 (31.2)	4 (25.0)		2 (12.5)	5 (31.3)	9 (56.2)	

Frequency and row percentages are reported.

a p-values obtained from chi-squared test.

b p-values obtained from Fisher’s exact tests.

Table 3 provides the bivariate associations between smoking and e-cigarette use restrictions and smoking and e-cigarette use behaviors. Dual users with complete (vs no) home smoking restrictions reported lower smoking intensity (i.e. fewer smoking days in the past 7 days; fewer cigarettes smoked per day), later time to the first cigarette, and higher e-cigarette use intensity (number of days using e-cigarettes in the past 7 days). Daily e-cigarette use was reported by more than half (50.4%) of dual users with complete restrictions versus 30.7% of those with no restrictions (p=0.025). In addition, among those with a complete home smoking restriction, 27.4% were predominantly using e-cigarettes and 41.0% predominantly smoking, whereas 10.7% and 66.7% of dual users with no home smoking restrictions were predominantly using e-cigarettes and predominantly smoking, respectively (p=0.005). Those with complete (vs no) smoking restrictions were more likely to be in a later stage of quitting smoking (i.e. contemplation or preparation). E-cigarette use restrictions were only significantly associated with the stage of quitting smoking (p=0.037). Similar to smoking restrictions, those who reported complete (vs no) e-cigarette use restrictions were more likely to be in a later stage of quitting smoking (i.e. contemplation or preparation).

**Table 3:** Bivariate associations between home restrictions and smoking and e-cigarette use behaviors, a crosssectional analysis of data from the Adult Consumers of Tobacco Study 2020–2021

*Cigarette and e-cigarette use* *characteristics*	*Home smoking restrictions*				*Home e-cigarette use restrictions*			
	*Complete* *restrictions* *(N=117)*	*Partial* *restrictions* *(N=58)*	*No* *restrictions* *(N=75)*	*p*	*Complete* *restrictions* *(N=32)*	*Partial* *restrictions* *(N=49)*	*No* *restrictions* *(N=169)*	*p*
Number of smoking days in the past 7 days, mean (SD)	5.5 (2.3)	6.1 (1.8)	6.6 (1.2)	0.003^a^	5.8 (2.0)	6.0 (1.9)	5.9 (1.9)	0.94^a^
Number of cigarettes per smoking days, mean (SD) (N=246)	10.7 (7.7)	14.3 (12.0)	16.3 (8.1)	<0.001^a^	10.5 (6.4)	13.3 (9.7)	13.7 (9.6)	0.20^a^
Time to the first cigarette (minutes), mean (SD) (N=245)	51.5 (93.4)	21.3 (25.7)	16.3 (30.3)	<0.001^a^	32.5 (41.1)	43.2 (107.0)	31.4 (58.9)	0.39^a^
Number of days using e-cigarettes in the past 7 days, mean (SD)	4.9 (2.5)	4.7 (2.2)	3.8 (2.5)	0.001^a^	4.3 (2.3)	4.5 (2.7)	4.6 (2.4)	0.83^a^
**E-cigarette use frequency**				0.025**^b^**				0.16
Some days	58 (49.6)	32 (55.2)	52 (69.3)		23 (71.9)	25 (51.0)	94 (55.6)	
Every day	59 (50.4)	26 (44.8)	23 (30.7)		9 (28.1)	24 (49.0)	75 (44.4)	
**Concurrent use behavior**				0.005**^b^**				0.69**^b^**
Predominant e-cigarette use	32 (27.4)	10 (17.2)	8 (10.7)		4 (12.5)	12 (24.5)	34 (20.1)	
Predominant smoking	48 (41.0)	27 (46.6)	50 (66.7)		19 (59.4)	22 (44.9)	84 (49.7)	
Equivalent use	37 (31.6)	21 (36.2)	17 (22.7)		9 (28.1)	15 (30.6)	51 (30.2)	
**Stage of change for quitting smoking**				0.005**^b^**				0.037**^c^**
Precontemplation	11 (9.4)	4 (6.9)	12 (16.0)		1 (3.1)	2 (4.1)	24 (14.2)	
Contemplation	65 (55.6)	34 (58.6)	54 (72.0)		19 (59.4)	28 (57.1)	106 (62.7)	
Preparation	41 (35.0)	20 (34.5)	9 (12.0)		12 (37.5)	19 (38.8)	39 (23.1)	

Frequency and column percentages are reported for categorical variables. N=250 unless otherwise indicated.

a p-values obtained from Kruskal-Wallis tests.

b p-values obtained from chi-squared test.

c p-value obtained from Fisher’s exact tests.

Results from the regression analyses are presented in Table 4. Those with complete (vs no) smoking restrictions had more than two times the odds of using e-cigarettes predominantly versus equivalent use or predominant smoking (AOR=2.60; 95% CI: 1.30–5.30), after adjusting for home e-cigarette use restrictions and other covariates. In addition, greater time to the first cigarette was associated with higher odds of predominant e-cigarette use (AOR=1.01; 95% CI: 1.0003–1.01), whereas female (vs male) dual users were less likely to be predominant e-cigarette use or equivalent use versus predominant smoking (AOR=0.53; 95% CI: 0.30–0.96). Complete e-cigarette use restrictions (vs no restrictions) were associated with lower odds of predominant e-cigarette use versus equivalent use or predominant smoking (AOR=0.36; 95% CI: 0.14–0.91). However, neither partial smoking restrictions nor partial e-cigarette restrictions were associated with concurrent use behavior. In the logistic regression, those with complete home smoking restrictions also had more than two times higher odds of using e-cigarettes daily compared to those who were allowed to smoke anywhere and at any time inside their houses (AOR=2.60; 95% CI: 1.21–5.49). Conversely, complete (vs no) e-cigarette use restrictions were associated with lower odds of daily e-cigarette use (AOR=0.28; 95% CI: 0.10–0.79). We also explored additional models examining interactions between home smoking restrictions and e-cigarette use restrictions, which were not significant for both outcomes.

**Table 4:** Association between concurrent use behaviors, e-cigarette use frequency and home smoking and e-cigarette use restriction status, a cross-sectional analysis of data from the Adult Consumers of Tobacco Study 2020–2021

*Variables*	*Concurrent use behavior ^a^*		*E-cigarette use frequency ^b^*	
	*AOR*	*95% CI*	*AOR*	*95% CI*
**Home smoking restrictions**				
No restrictions ®	1		1	
Partial restrictions	1.46	0.68–3.13	1.48	0.65–3.36
Complete restrictions	2.60**	1.30–5.30	2.60*	1.21–5.49
**Home e-cigarette use restrictions**				
No restrictions ®	1		1	
Partial restrictions	0.70	0.34–1.43	0.79	0.36–1.74
Complete restrictions	0.36*	0.14–0.91	0.28*	0.10–0.79
**Time to the first cigarette** (minutes)	1.01*	1.00–1.01c	1.00	0.99–1.01
**Age** (years)	0.98	0.97–1.01	0.98	0.95–1.02
**Gender**				
Male ®	1		1	
Female	0.53*	0.30–0.96	0.60	0.32–1.13
**Income** (US$)				
<25000 ®	1		1	
25000–49000	0.83	0.43–1.63	0.61	0.30–1.26
50000–99000	1.41	0.69–2.87	1.33	0.61–2.91
≥100000	0.64	0.12–3.39	1.14	0.18–7.22
**Education level**				
Lower than high school diploma ®	1		1	
High school diploma or equivalent	1.34	0.32–5.58	1.35	0.31–5.89
Some colleges, no degree	0.73	0.17–3.08	0.89	0.20–3.87
Bachelor’s or higher	1.27	0.29–5.51	1.12	0.25–5.07
**Race/ethnicity**				
White, non-Hispanic ®	1		1	
Black, non-Hispanic	0.97	0.41–2.31	1.41	0.55–3.63
Hispanic, any race or other race	1.16	0.53–2.58	1.38	0.59–3.23
**Other members own e-cigarettes at home**				
No ®	1		1	
Yes	1.53	0.86–2.71	1.66	0.90–3.07
**Stage of change for quitting smoking**				
Precontemplation ®	1		1	
Contemplation	2.59	0.91–7.39	1.89	0.66–5.41
Preparation	8.04***	2.59–24.89	5.14**	1.60–16.49
Model fit	N=241, Pseudo R^2^=0.13		N=241, Pseudo R^2^=0.128	

Nine observations excluded from the regressions: 4 reported non-cis gender, and 5 missing data on the ‘time to the first cigarette’ variable.

a Ordinal regression with dual use coded as: 1=predominant e-cigarette use, 2=equivalent use, 3=predominant smoking. The p-value for the likelihood-ratio test of proportionality odds=0.38, indicating the proportional odds assumption is reasonable.

b Logistic regression with e-cigarette use frequency coded as 1=using every day or 0=using on some days.

c The exact 95% confidence interval: 1.0003–1.0103.

* p<0.05,

** p<0.01,

*** p<0.001. ® Reference categories.

## DISCUSSION

To the best of our knowledge, this is one of the first studies to examine the associations between home smoking and e-cigarette use restrictions and concurrent cigarette and e-cigarette use behaviors among a sample of newly established dual users. We found that a few dual users had both complete home smoking and e-cigarette use restrictions, and home smoking restrictions were more common than e-cigarette use restrictions. In addition, as we hypothesized, dual users with complete home smoking restrictions versus no restrictions were more likely to use e-cigarettes more frequently than smoking cigarettes and to use e-cigarettes daily. Complete home e-cigarette use restrictions were associated with a lower likelihood of daily e-cigarette use (vs some days) and a lower likelihood of predominant e-cigarette use (vs equivalent use or predominant smoking). However, neither partial home smoking restriction nor partial home e-cigarette use restrictions were associated with concurrent use behaviors and e-cigarette use frequency.

A study using Wave 4 (2016–2018) Population Assessment of Tobacco and Health Study data reported that almost 70% of US adults did not allow the use of three types of tobacco products (cigarettes, e-cigarettes, smokeless tobacco) in their homes^27^. In addition, data from a 2017 internet-based nationally representative survey (n=4107) indicated that 56.8% of US adults did not allow using e-cigarettes in their homes^28^. In the current study, 46.8% of dual users reported having complete smoking restrictions, 12.8% complete e-cigarette use restrictions, and 11.2% both complete smoking and e-cigarette use restrictions. Another study among parents reported that 63% of dual users of cigarettes and e-cigarettes had complete home smoking restrictions vs 26.3% complete e-cigarette use restrictions^29^. Although smoking restrictions are generally stricter when having children living in the home, the small proportion of dual users in the current study who reported having both smoking and e-cigarette use restrictions suggests that there is a need to continue monitoring the temporal trends in adopting home smoking and e-cigarette use restrictions in the general population. Our findings also highlight the opportunity to educate tobacco product users and non-users about the harmful effects of secondhand smoke and aerosols from e-cigarettes on non-users^30^ to increase the adoption of home smoking and e-cigarette use restrictions.

We also found that dual users who reported having complete smoking restrictions were more likely to use e-cigarettes predominantly (vs predominantly smoking) and to use e-cigarettes daily (vs non-daily). Because e-cigarettes can be substitutes for cigarettes, many dual users who have complete home smoking restrictions may use e-cigarettes more often to satisfy their nicotine demand, especially since many people were working from home due to the COVID-19 pandemic. Numerous studies have shown that daily e-cigarette use is critical to successfully transition from dual use to smoking cessation^31-33^. Thus, it is possible that home smoking restrictions may have an indirect (mediated through concurrent use behavior) positive effect on cessation outcomes among dual users, especially those who want to use e-cigarettes to quit smoking. However, this potential positive effect will not be realized if concurrent use behavior is not followed by a complete transition away from cigarettes and/or a complete quit of both products eventually. Future studies, particularly those using longitudinal designs, would be useful to examine this effect. In addition, we did not observe significant associations between partial restrictions and concurrent use behavior or e-cigarette use frequency of dual users, which is consistent with a previous study reporting that partial home smoking restrictions were no better than no restrictions with regard to cigarettes per day and time to the first cigarette, and may cause an increase in urges to smoke in the morning^14^. Another study among a low-income population found that partial restrictions posed challenges to the enforcement of home smoking restrictions, as participants with partial (vs complete) restrictions reported higher rates of smoking in all rooms except children’s bedrooms^34^. However, the concept of partial restrictions is ambiguous, and it is difficult to measure and control for the variability in the degree of implementation and enforcement of such restrictions.

A study using data from the 2014–2018 National Health Interview Survey^35^ reported smoke-free worksite laws were associated with a decrease in the likelihood of current smoking and recent e-cigarette use and an increase in the likelihood of smoking cessation, but adding e-cigarette use restrictions to smoke-free worksite policies was not associated with further reductions in recent e-cigarette use and counteracted over half of the estimated association with current smoking relative to smoke-free policies alone. Even though our study examined smoke-free policies in home environments, we observed a significant association between home e-cigarette use restrictions and e-cigarette use frequency and concurrent use behavior. Aerosol from e-cigarettes can contain harmful substances, including cancercausing chemicals and tiny particles^36^. Thus, adopting e-cigarette home restrictions should also be promoted widely. We did not observe a significant effect of the interaction between home smoking restrictions and e-cigarette use restrictions on concurrent use behaviors. However, future studies with larger samples are needed to confirm this finding and more closely investigate how home smoking and e-cigarette use restrictions may jointly affect smoking and e-cigarette use behaviors.

### Limitations

Our findings should be interpreted in light of the study’s limitations. First, this sample of dual users was demographically diverse but small, restricted to adults aged ≥21years, and recruited online mostly through Facebook and Instagram, limiting generalizability to those aged ≥21 years and with internet access and social network accounts. Related, selection bias could have impacted findings, and the small sample size precluded analyses examining other subgroups of dual users (i.e. daily dual users or non-daily dual users) and other covariates (i.e. race/ethnicity, gender identity). In addition, our classification of dual user subgroups was based on daily or some-days frequency of smoking and e-cigarette use; we were not able to account for the intensity or average amount of nicotine consumed per day. Thus, it is possible that a participant might consume more nicotine or use e-cigarettes more intensely even though they reported smoking every day and using e-cigarettes some days.

Dual users in our study were those who recently initiated/re-initiated using e-cigarettes; there would be more variability in their e-cigarette use behaviors caused by other factors that we may fail to control for in our analyses. Data collection occurred during the COVID-19 pandemic, and as such, some findings may not generalize to the post-COVID period. Finally, assessing causal relationships between smoking and e-cigarette use restrictions and concurrent use behaviors was precluded due to the cross-sectional design of the study.

## CONCLUSIONS

In a sample of dual users of cigarettes and e-cigarettes recruited online, we found that complete smoking and e-cigarette use restrictions were not widely adopted in their homes, indicating a need for monitoring and encouraging the adoption of smoke-free home policies, and the opportunities to educate tobacco product users about the harmful effects of secondhand smoke and aerosols from e-cigarettes to increase their support in implementing voluntary smoking and e-cigarette use restrictions in their homes. In addition, we found a significant association between home smoking restrictions and concurrent use behavior and e-cigarette use frequency among dual users, suggesting that home smoking restriction may have incentivized people who smoke cigarettes to substitute smoking with e-cigarette use.

## Data Availability

The data supporting this research are available from the authors on reasonable request.
